# The effect of temperature on pyrolysis products during oil shale thermal decomposition

**DOI:** 10.1038/s41598-025-11050-6

**Published:** 2025-07-18

**Authors:** Fajun Zhao, Zian Yang, Lei Zhang, Changjiang Zhang, Tianyu Wang, Hong Zhang

**Affiliations:** 1 Northeast Petroleum University Key Laboratory of Improving Oil and Gas Recovery,Ministry of Education, Daqing, 163318 China; 2 State Key Laboratory of Continental Shale Oil, Daqing, 163712 China; 3 Exploration and Development Research Institute of Daqing Oilfield Co Ltd, Daqing, 163712 China

**Keywords:** Oil shale, Pyrolysis, Temperature, Shale oil, Mechanism analysis, Energy science and technology, Engineering

## Abstract

This study systematically investigates the effect of temperature on product distribution and reaction mechanisms during the pyrolysis of oil shale through thermogravimetric analysis (TG) and fixed-bed pyrolysis experiments. The results indicate that the oil shale pyrolysis process can be divided into three stages: the low-temperature stage, dominated by water evaporation; the mid-temperature stage (400–650 °C), which is the primary stage for organic matter decomposition; and the high-temperature stage (> 650 °C), characterized by secondary cracking of heavy components and decomposition of mineral residues. The study reveals that temperature significantly influences the generation and distribution of gas, liquid, and solid products. Gas yield increases markedly under high-temperature conditions, especially for H_2_ and CH_4_. The shale oil yield reaches its peak at mid-temperatures (400–500 °C), but decreases at higher temperatures due to secondary cracking reactions. The fixed carbon content of the semi-coke decreases with increasing temperature, while the mineral components decompose into porous oxides at high temperatures. Kinetic analysis demonstrates that primary reactions drive the mid-temperature pyrolysis, whereas secondary reactions enhancing gas production become dominant at high temperatures. This study elucidates the regulatory mechanisms of temperature on oil shale pyrolysis behavior, providing guidance for the targeted optimization of specific products, such as gases, shale oil, or semi-coke. The findings offer scientific and technical references for optimizing pyrolysis processes and improving the efficient utilization of unconventional energy resources.

## Introduction

With the continuous growth of global energy demand and the gradual depletion of traditional fossil fuels, the development and utilization of unconventional energy sources have received significant attention. Oil shale, as an important unconventional resource, has abundant reserves and widespread distribution, making it one of the key areas for future energy development^1,2^. Pyrolysis technology is the core method for converting oil shale resources. Through high-temperature treatment, the organic matter in oil shale is decomposed into gas, liquid, and solid products. The liquid product (shale oil) can be further refined into petroleum substitutes, the gaseous product can be used for energy supply, and the semi-coke can be utilized in construction materials or mineral recovery^3,4^.

Temperature, as a key control parameter in the pyrolysis process, not only directly influences the rate and characteristics of the pyrolysis reaction but also significantly affects the production and distribution of the resulting products^5^. Studies have shown that temperature variations play an essential role in the release of gaseous components, the yield and composition of shale oil, and the physicochemical properties of semi-coke. For example, at lower temperatures, the pyrolysis reaction predominantly produces liquid products, whereas higher temperatures favor the generation of gaseous products. Moreover, secondary cracking reactions may occur at high temperatures, leading to an increase in light fractions of shale oil while releasing more combustible gases^6–10^. Although existing research has explored the effect of temperature on the pyrolysis of oil shale, most of these studies focus on specific temperature ranges or specific products, lacking systematic analysis of the distribution and reaction mechanisms of gaseous, liquid, and solid three-phase products during the pyrolysis process of oil shale from low to high temperatures. In addition, there is still insufficient research on how temperature affects reaction pathway selection through kinetic parameters such as activation energy.

Therefore, this study combines thermogravimetric analysis (TG) and retort pyrolysis experiments to systematically explore the segmented regulation mechanism of temperature in the entire process of oil shale pyrolysis and its influence on the distribution and characteristics of gaseous, liquid, and solid products^11,12^. In addition, the contribution rate of reaction pathways in different temperature ranges was quantified by applying a multi-step kinetic model. This study not only provides a scientific basis for optimizing the pyrolysis process of oil shale, but also lays a theoretical foundation for the efficient utilization of unconventional energy.

## Experimental section

### Experimental materials

The oil shale samples from a certain block of Daqing Oilfield selected in this study were dried, ground, and sieved, and particles with a particle size of 1–3 mm were selected for analysis. The characteristics of the oil shale were determined through approximate analysis and elemental analysis, where the elemental analysis results were presented on a dry basis, i.e. after removing the sample moisture, to more accurately reflect the actual content of organic and inorganic components. The analysis results are shown in Table 1.

**Table 1:** Elemental Composition and Mineral Content of Oil Shale Samples.

Elemental analysis (wt%)		Proximate analysis (wt%)		Mineral composition (wt%)			
C	13.44	Moisture	3.8	Quartz	28	Siderite	0.5
H	0.46	Volatile matter	22.4	Feldspar	5.2	Pyrite	1.7
N	0.38	Ash	84.2	Clay minerals	23.2		
S	0.58	Fixed carbon	1.6	Calcite	33.8		
O	0.94	–	–	Dolomite	7.6		

### Experimental apparatus and conditions

#### Thermogravimetric analysis (TG) experiment

Thermogravimetric analysis was performed using a TGA instrument (such as the NETZSCH TG 209). The main experimental parameters were set as follows:

Sample weight: approximately 10 mg; Heating rates: set to 5 °C/min, 10 °C/min, 15 °C/min, 20 °C/min, and 25 °C/min; Temperature range: from room temperature to 950 °C; Atmosphere: high-purity nitrogen gas (N_2_) with a flow rate of 50 mL/min to prevent sample oxidation; Data collection: real-time recording of the sample mass change with temperature (TG curve) and the differential mass loss curve (DTG curve).

#### Retort pyrolysis experiment

**Fig. 1:**
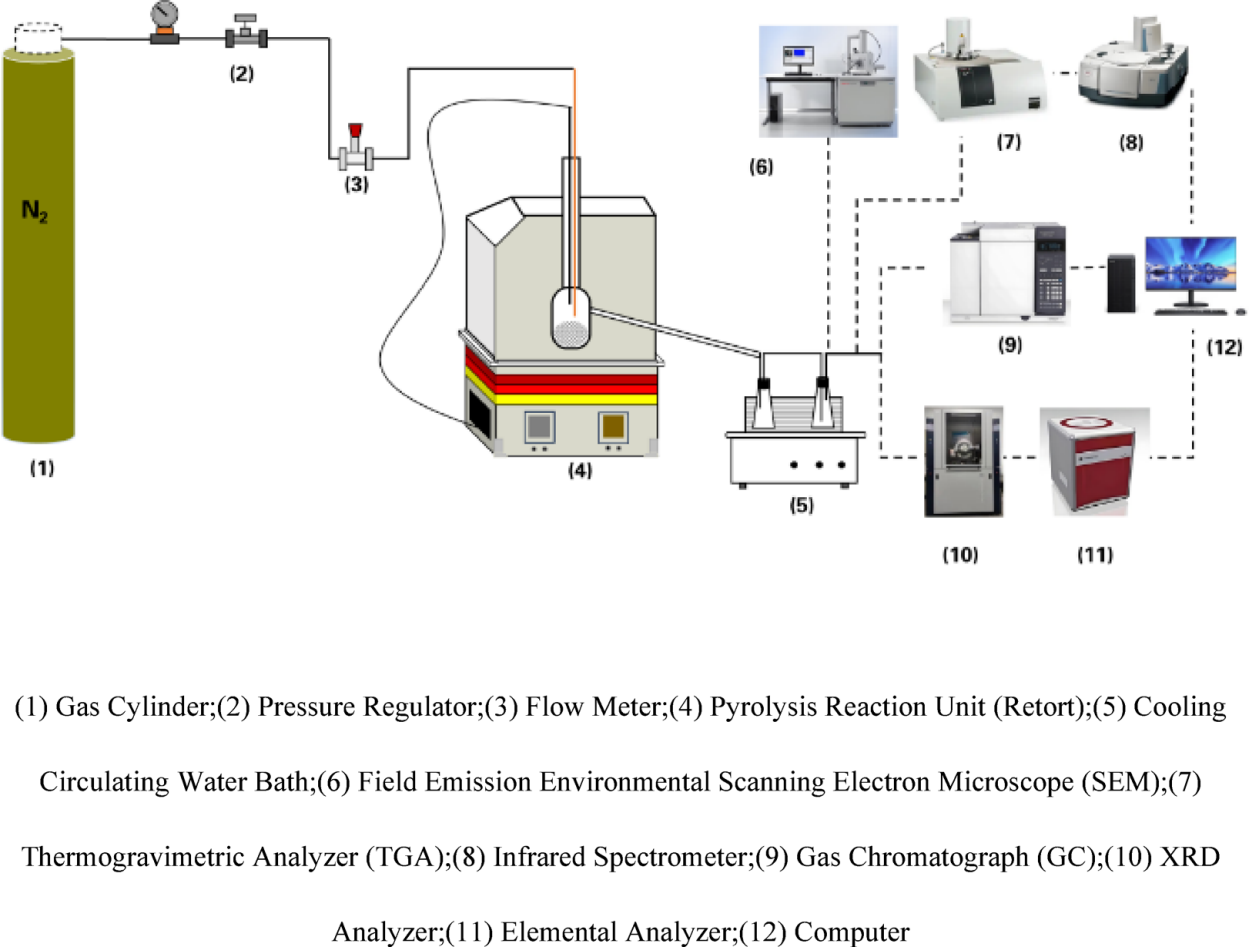
Schematic diagram of oil shale pyrolysis experimental apparatus and analytical methods.

The retort pyrolysis experiments were conducted using a small fixed-bed pyrolysis system to simulate industrial conditions. The experimental apparatus, as illustrated in Fig. 1, included a reactor, an electric heating system, a condensation system, and a gas collection system. The reactor utilized a fixed-bed design with an inner diameter of 50 mm and a length of 300 mm, fabricated from 316 stainless steel. The experiments were conducted at atmospheric pressure (1 atm), maintained by a continuous nitrogen flow of 50 mL/min. The heating system comprised an electric furnace equipped with PID temperature control, achieving a temperature accuracy of ± 2 °C. The experimental conditions were as follows:

Sample weight: 50 g of oil shale per test; Heating conditions: final temperatures were set to 400 °C, 450 °C, 500 °C, 550 °C, and 650 °C, with a fixed heating rate of 20 °C/min; Condensation system: used to collect liquid products (shale oil and moisture); Gas collection: gas products were collected using gas bags for component analysis.

### Product analysis methods

#### Gas product analysis

The gas products generated during the pyrolysis process were collected using gas bags and analyzed using gas chromatography (GC) to determine the main components, including H_2_, CO_2_, and CH_4_. The analysis conditions were as follows:

Chromatographic column: specialized gas separation column; Carrier gas: high-purity helium (He);Detector: thermal conductivity detector (TCD).

#### Liquid product analysis

The liquid products collected by the condensation system were divided into shale oil and water. The yield of each was measured by weighing. The chemical composition of the shale oil was analyzed using Fourier Transform Infrared Spectroscopy (FTIR) and Gas Chromatography (GC). FTIR analysis was performed using a Nicolet iS10 spectrometer with a spectral range of 4000–400 cm^−1^ and a resolution of 4 cm^−1^. Solid samples were prepared using the KBr pellet method, and the data was processed using OMNIC software. Gas chromatography analysis was performed using Agilent 7890B, equipped with a thermal conductivity detector (TCD) and a flame ionization detector (FID). The chromatographic column is HP-LOT/Q capillary column (30 m × 0.53 mm), the carrier gas is helium (He), and the flow rate is set at 2.0 mL/min. The oven temperature program is set to an initial temperature of 40 °C and held for 5 min, followed by heating up to 200 °C at a rate of 10 °C/min.

#### Semi-coke analysis

The physical and chemical properties of the semi-coke after pyrolysis were analyzed as follows:

The mass of the semi-coke was measured to calculate the yield; The primary mineral composition of the residue was analyzed using X-ray Diffraction (XRD);The pore morphology and microstructural changes on the residue surface were observed using Scanning Electron Microscopy (SEM). X-ray diffraction analysis (XRD) was performed using Rigaku SmartLab, equipped with a Cu–Kα radiation source, under operating conditions of 40 kV and 40 mA, with a scanning range of 5–80° 2θ. Scanning electron microscopy (SEM) analysis was performed using Hitachi SU8010, with an acceleration voltage set to 5 kV, and the sample was treated with gold plating to enhance conductivity.

### Data processing

The yields of gas, liquid, and solid products were calculated using the following formulas:

1 $$Gas \; yield \; \left( \% \right) = \frac{Mass \; of \; gas \; product}{{Initial \; mass \; of \; sample}} \times 100$$

2 $$Liquid \; yield \; \left( \% \right) = \frac{Mass \; of \; liquid \; product}{{Initial \; mass \; of \; sample}} \times 100$$

3 $$Solid \; yield \; \left( \% \right) = \frac{Mass \; of \; solid \; residue}{{Initial \; mass \; of \; sample}} \times 100$$

Based on the TG/DTG curves, the initial temperature, the temperature at the maximum weight loss rate, and the final temperature were determined. For kinetic analysis**,** both model-free and model-fitting methods were applied to calculate the pyrolysis reaction’s kinetic parameters, including the activation energy (Ea) and the pre-exponential factor (A).

## Experimental results and discussion

### Effect of temperature on pyrolysis behavior

**Fig. 2:**
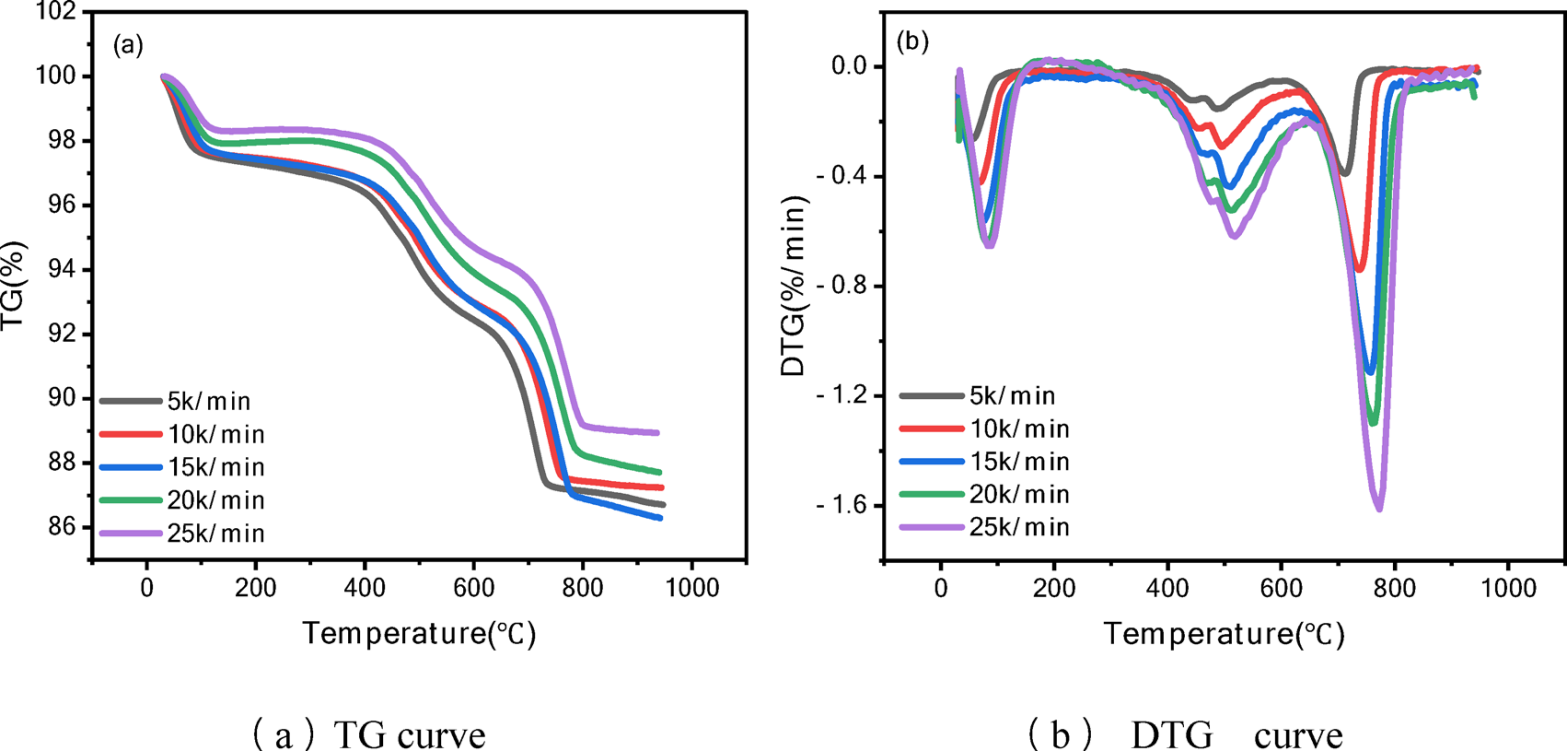
TGA-DTG curves of oil shale samples at different heating rates under a nitrogen atmosphere.

Through thermogravimetric analysis (TG) experiments, the mass change curves (TG) and weight loss rate curves (DTG) of oil shale at different temperatures were obtained, as shown in Fig. 2. The experimental results showed that:

From Fig. 2 TG curve (a), it can be concluded that as the temperature increases, the TG curve shows a gradual decrease in the mass of the oil shale sample, indicating thermal decomposition and the release of volatile substances. As the heating rate increases, the TG curve shifts to the right (higher temperature region), suggesting that a higher heating rate requires a higher temperature for thermal decomposition to occur. This is due to the thermal lag effect.

Low-temperature region (0–200 °C): The curve shows minimal changes, mainly due to the evaporation of moisture and a small amount of volatile matter. Medium-temperature region (200–650 °C): Significant mass loss occurs, which is the primary temperature range for the pyrolysis of organic matter in oil shale. High-temperature region (650–950 °C): The curve levels off, indicating that pyrolysis is nearly complete, leaving ash and inorganic residues.

From the DTG curve (b), it can be observed that the DTG curve exhibits multiple peaks, representing different stages of mass loss. A prominent peak appears in the range of approximately 400–650 °C, corresponding to the rapid pyrolysis of organic matter in oil shale, during which a large amount of volatile components is released. Smaller peaks at lower and higher temperatures correspond to moisture evaporation and the decomposition of certain inorganic carbonates, respectively. As the heating rate increases, the position of the DTG peaks shifts toward higher temperatures, and the peak intensity (rate) also increases. This indicates that the pyrolysis reaction rate accelerates with an increasing heating rate. The phenomenon of DTG peak temperature shifting with increasing heating rate can be attributed to three mechanisms: firstly, the thermal hysteresis effect leads to a temperature gradient inside the sample during rapid heating, causing the actual decomposition temperature to be higher than the detection value; Secondly, according to the Arrhenius kinetic relationship, higher rates require higher temperatures to achieve the same reaction rate; Finally, the heating rate affects the competitive relationship between primary cracking and secondary reactions (such as coke deposition) by regulating the retention time of volatiles. These factors work together to cause the DTG peak temperature to shift towards higher temperatures.

To further analyze the pyrolysis behavior of oil shale, key combustion characteristic parameters were extracted from the thermogravimetric (TGA) and derivative thermogravimetric (DTG) curves. These parameters include the ignition temperature (*T*_*i*_), peak temperature (*T*_*p*_), burnout temperature (*T*_*f*_), and the mass loss ratio. These parameters reflect the thermochemical characteristics of the combustion process, including the initiation, main reaction, and final completion stages. Specifically, the ignition temperature (*T*_*i*_) marks the starting point of significant weight loss in the sample, which is reflected on the TG curve as the first obvious mass loss starting position, and corresponds to the temperature at which the DTG curve begins to deviate significantly from the baseline. The peak temperature (*T*_*p*_) reflects the peak of the sample weight loss rate, which is the temperature point at which the DTG curve reaches the maximum weight loss rate. The burnout temperature (*T*_*f*_) represents the endpoint at which the sample weight loss process is essentially completed, usually corresponding to the temperature point on the TG curve when the mass loss tends to stabilize. The combustion characteristic parameters extracted from the experimental data at different heating rates are listed in Table 2.

**Table 2:** Pyrolysis characteristic parameters of oil shale at different heating rates.

Heating rate (°C/min)	*T*_*i*_ (°C)	I *T*_*p*_ (°C)	II *T*_*p*_ (°C)	III *T*_*p*_ (°C)	*T*_*f*_ (°C)	Total mass loss (%)
5	329	51	481	713	776	13.3
10	348	69	494	736	789	12.8
15	358	71	507	754	799	13.7
20	362	77	511	765	817	12.3
25	383	83	512	773	829	11.1

As shown in Table 2, the heating rate has a significant impact: as the heating rate increases, the ignition temperature, peak temperatures, and burnout temperature all shift to higher temperature regions. An increase in the heating rate causes the total mass loss to decrease slightly, indicating that the sample decomposes incompletely at higher heating rates. As the heating rate increased from 5 to 25 °C/min, the total mass loss of the sample decreased from 13.3 to 11.1%, indicating incomplete decomposition of oil shale at higher heating rates. This phenomenon is mainly attributed to the internal heat transfer delay and temperature gradient caused by thermal hysteresis effect^5,8^, which leads to insufficient thermal decomposition of internal particles, and rapid heating shortens the residence time of organic matter in the critical temperature range, hindering the secondary cracking of heavy components and mineral decomposition^6^. In addition, primary cracking reactions dominate at high heating rates, while secondary reactions that require higher activation energies (such as mineral decomposition and coke formation) are partially suppressed due to insufficient reaction time^13^. These factors work together to lead to a decrease in decomposition efficiency at high heating rates. Temperature is the main driving factor for the pyrolysis of organic matter in oil shale, and a higher final temperature promotes the complete decomposition of organic matter.

### Effect of temperature on gaseous products

**Fig. 3:**
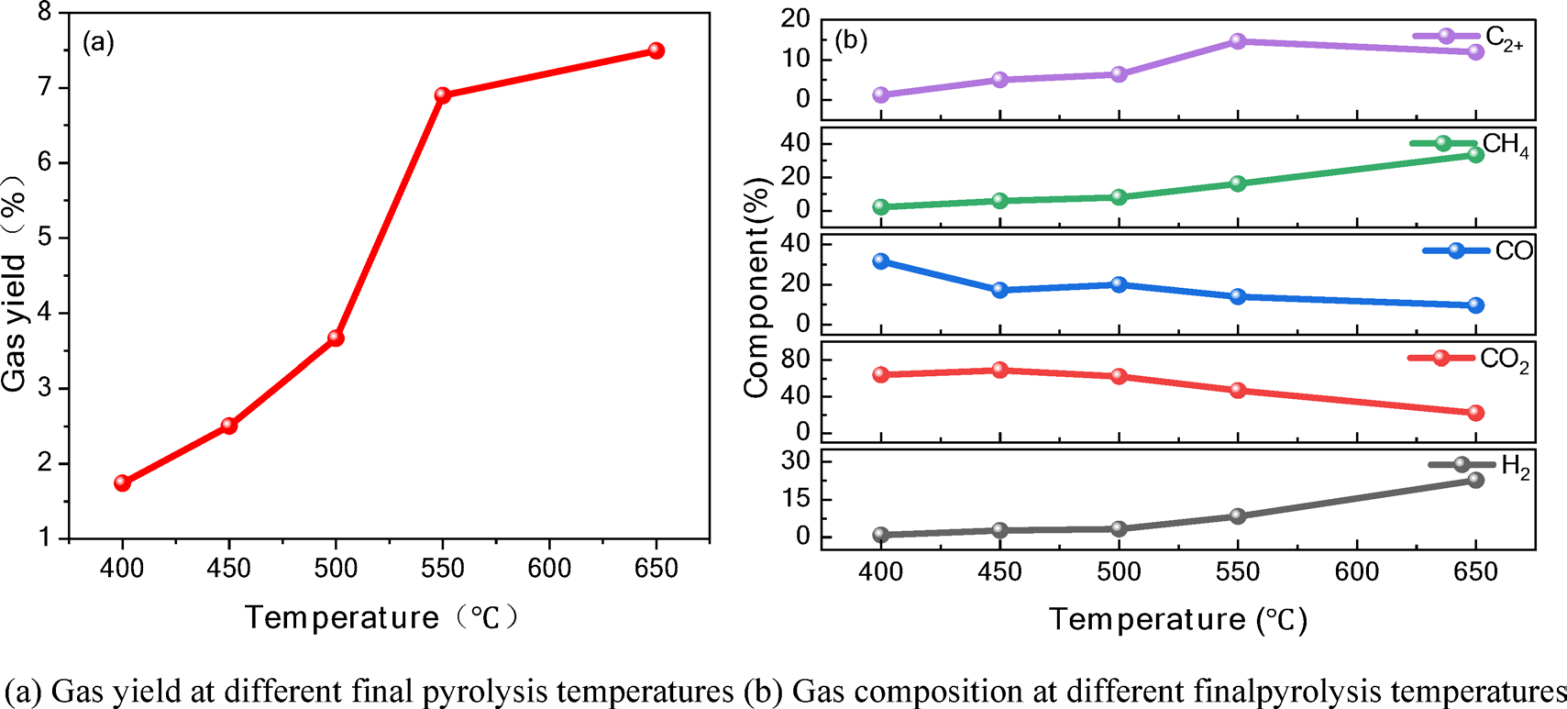
Variations in gas release at different final pyrolysis temperatures.

During the pyrolysis of oil shale, temperature has a significant influence on the release patterns of gaseous products. Figure 3 shows the variations in gas release at different final pyrolysis temperatures.

From Fig. 3a, it can be observed that as the temperature increases, the total gas yield increases. High temperatures promote the deep cracking of heavy components and enhance the generation of combustible gases such as H_2_ and CH_4_. This indicates that temperature control can significantly influence the production rate and compositional ratio of gaseous products.

In the retort pyrolysis experiment, the gas components (H_2_, CO_2_, CH_4_, etc.) generated at different temperatures were analyzed using gas chromatography. The results showed the following trends:

H_2_ content gradually increased from a low proportion at 400 °C to a maximum at 650 °C, reaching nearly 70%. This indicates that as the temperature increases, the pyrolysis reaction of organic matter intensifies, and the release of H_2_ significantly increases, becoming the main product of high-temperature pyrolysis. This phenomenon is mainly attributed to the condensation dehydrogenation reaction of aromatic hydrocarbons and the free radical recombination generated during the β—chain cleavage process of fatty chains. These processes not only promote the generation of hydrogen, but also reflect the complex chemical change mechanisms during pyrolysis. In addition, pyrite (FeS_2_) acts as a catalyst to promote the dehydrogenation reaction of organic matter, helping to remove hydrogen atoms from hydrocarbons, promote the formation of aromatic hydrocarbons, and increase H_2_ production, playing a key regulatory role in the type and distribution of pyrolysis products.

CO_2_ is predominantly released at lower temperatures (450 °C) and then gradually decreases. The generation of CO_2_ during oil shale pyrolysis originates from multiple reaction pathways involving both inorganic and organic components. The inherent carbonate minerals in the inorganic matrix (particularly calcite and dolomite) undergo thermal decomposition reactions in the medium–high temperature range (400–650°C) to produce CO_2_^14^. Simultaneously, carboxyl functional groups in the organic kerogen undergo decarboxylation reactions during the initial pyrolysis stage (300–400 °C), while hydroxyl and carbonyl groups generate CO or CO_2_ through thermal cracking or rearrangement reactions at higher temperatures^4^. Furthermore, primary pyrolysis products (such as CO and CH_4_) can further promote CO_2_ generation through gas-phase reactions or secondary reactions with mineral oxides, particularly under sustained thermal conditions.

CO content increases with temperature, reaching a significant proportion at high temperatures (650 °C). This suggests that carbon oxidation and cracking reactions intensify at higher temperatures, leading to increased CO production.

CH_4_ content is relatively low at 400 °C but increases significantly as the temperature rises to 550–650 °C, indicating a trend of methane release during the pyrolysis of organic matter. The generation of CH_4_ is closely related to the release of methyl radicals, mainly from the cleavage of fatty side chains and the demethylation of methyl aromatic hydrocarbons, which together promote the formation of methane. In addition, dolomite (MgCa(CO_3_)_2_) decomposes into MgO, CaO, and CO_2_ during pyrolysis, where CO_2_ interacts with free radicals or intermediates generated by pyrolysis through reduction reactions to promote the formation of CH_4_, while calcium oxide and magnesium oxide act as alkaline catalysts to neutralize acidic substances and optimize the environment for methane formation.

C_2_ and higher hydrocarbons (C_2_H_4_, C_2_H_6_, C_3_H_8_, etc.) are primarily released during the mid-temperature stage, but their content gradually decreases at higher temperatures. This reflects the decomposition trend of long-chain organic compounds.

### Effect of temperature on liquid products

**Fig. 4:**
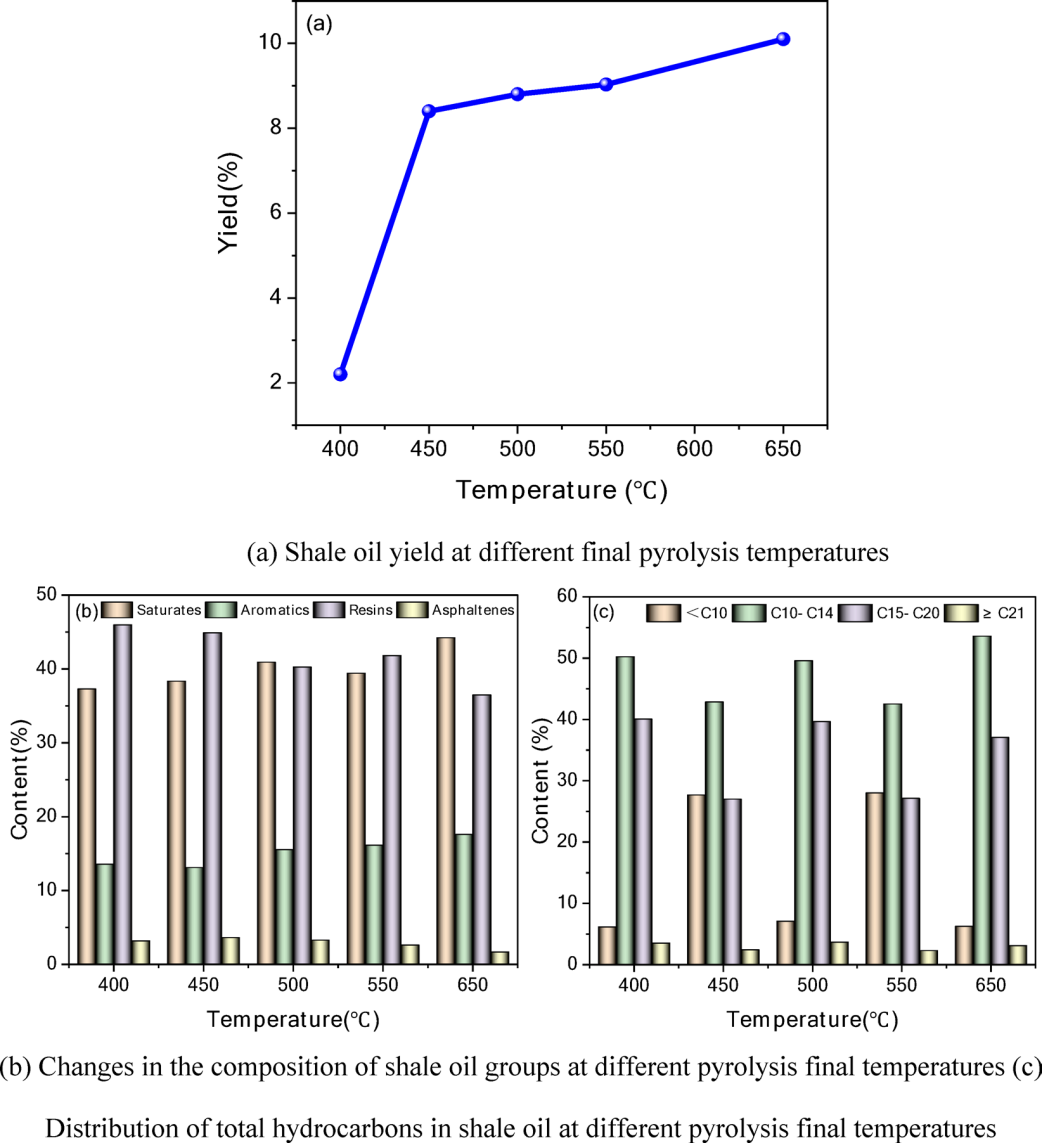
Changes in shale oil yield and composition at different pyrolysis final temperatures.

By studying the effect of pyrolysis conditions on shale oil yield and composition, process parameters can be optimized to improve the yield and quality of shale oil. To determine the optimal pyrolysis temperature range for maximizing shale oil yield, experiments were conducted to analyze the yield and compositional variations of shale oil at different final pyrolysis temperatures, as shown in Fig. 4.

From Fig. 4a, it can be observed that as the pyrolysis temperature increases, the oil yield increases significantly but tends to level off at higher temperatures. At low temperatures, the organic matter in oil shale is not completely decomposed, resulting in a lower shale oil yield. In the medium to high-temperature range (450–650 °C), the organic matter undergoes progressive pyrolysis, releasing a large amount of oil, thereby significantly increasing the shale oil yield. However, at excessively high temperatures, although the oil yield continues to rise, secondary cracking may occur, leading to an increase in light fractions and a gradual increase in coke formation.

From Fig. 4b, it can be seen that with increasing temperature, the content of alkanes and aromatics increases, while the proportion of resins and asphaltenes decreases. At 400 °C, the content of heavy components (colloids and asphaltenes) is relatively high, indicating a lower degree of organic matter cracking. At **650 °C**, the content of alkanes reaches a high level, suggesting that high temperatures promote the cracking of heavy components, making the oil lighter in composition.

From Fig. 4c, at low temperatures (400 °C), the content of C21 + (heavy components) is relatively high, while the content of light components (< C10 and C10–C14) is relatively low. As the temperature increases to 650 °C, the content of < C10 and C10–C14 increases, showing that light hydrocarbons gradually dominate. The C21 + heavy components decrease, indicating that high temperatures facilitate the thermal cracking of heavy oil.

By controlling the pyrolysis temperature, the yield and quality of shale oil can be effectively regulated. Selecting an appropriate temperature helps increase the yield of light oil and optimize the pyrolysis process.

### Effect of temperature on semi-coke

#### Semi-coke yield

**Fig. 5:**
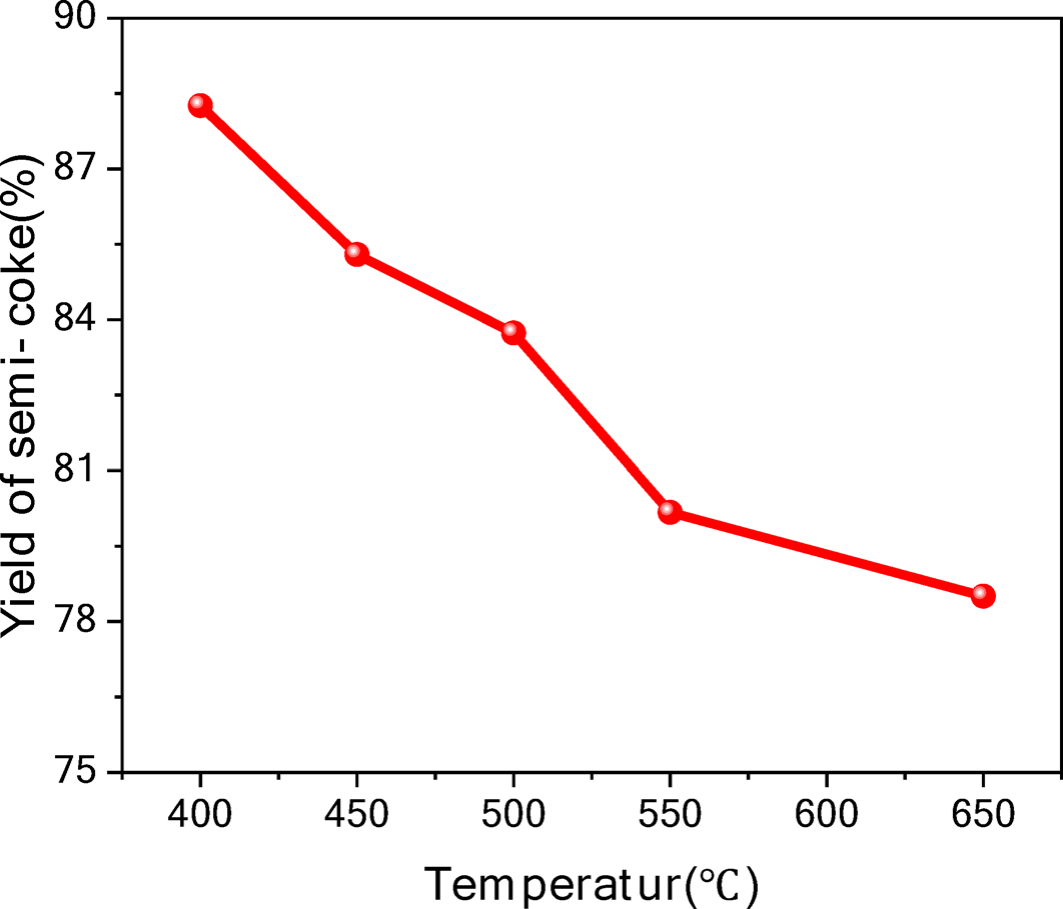
Effect of different final pyrolysis temperatures on semi-coke yield.

As illustrated in Fig. 5: Effect of different final pyrolysis temperatures on semi-coke yield, the semi-coke yield decreased progressively from 88 to 78% as the pyrolysis temperature increased from 400 to 650 °C. At lower temperatures (400–450 °C), the pyrolysis reaction of organic matter was relatively weak, resulting in only partial release of volatile components and a higher semi-coke yield. As the temperature increased (450–550 °C), the pyrolysis of organic matter in oil shale intensified, releasing significant amounts of volatile substances and reducing the semi-coke. When the temperature reached 650 °C, further cracking of the carbonaceous material in the semi-coke occurred, leading to a further decrease in the semi-coke yield.

#### Mineral composition

**Fig. 6:**
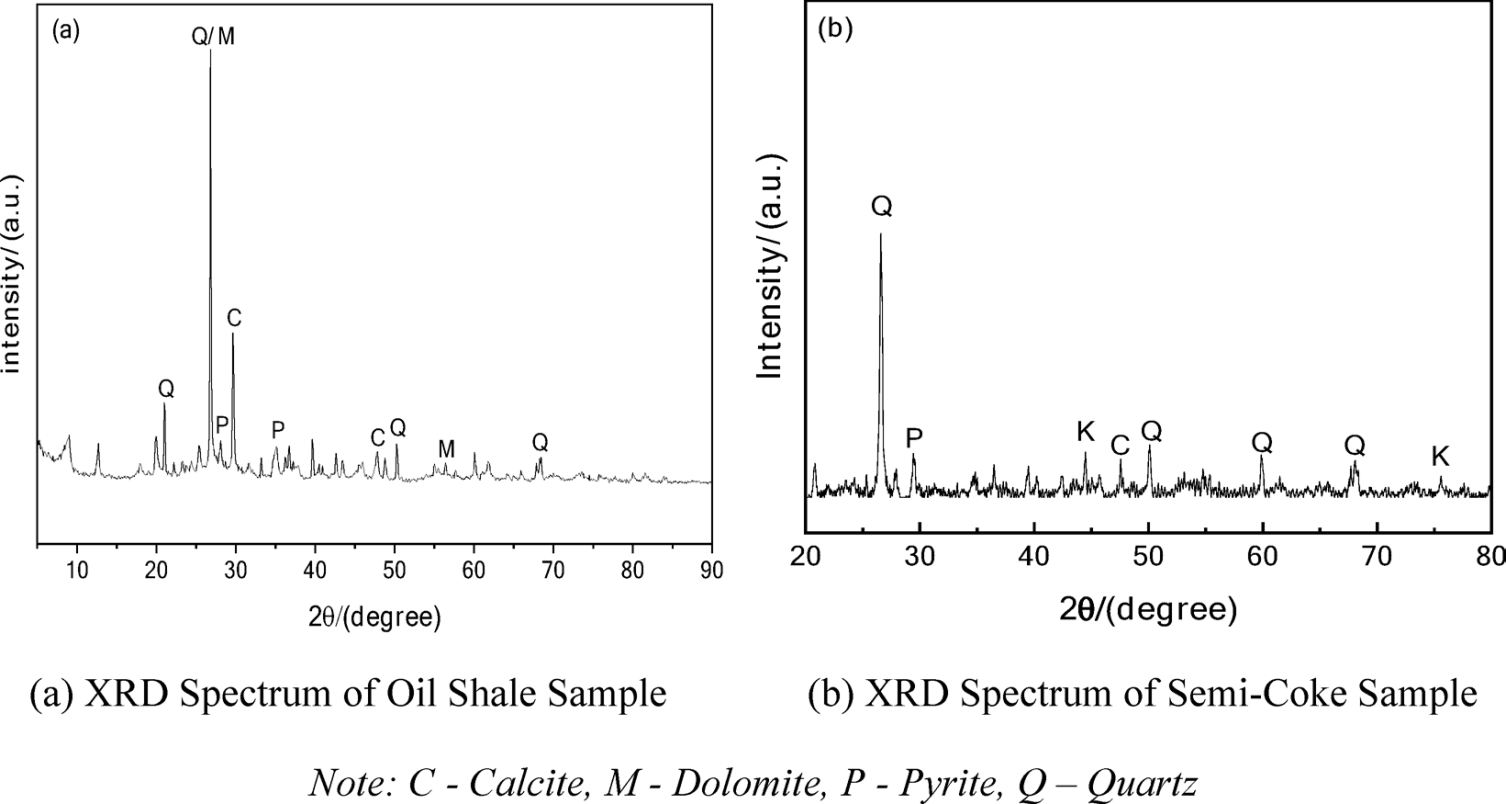
XRD spectra of oil shale and semi-coke samples. *Note* C—Calcite, M—Dolomite, P—Pyrite, Q – Quartz

To investigate the changes in mineral composition and crystal structure during the pyrolysis process, X-ray diffraction (XRD) analysis was conducted on both the oil shale samples and the semi-coke samples obtained at 650 °C. The findings are presented in Fig. 6.

The XRD spectrum of the oil shale sample (Fig. 6) shows: A pronounced quartz (Q) characteristic peak, indicating that quartz is one of the primary mineral components. Clear dolomite (M) characteristic peaks, suggesting that dolomite is present in significant proportions in the oil shale sample. High-intensity calcite (C) peaks, showing that calcite is an important carbonate mineral in the oil shale sample. The presence of pyrite (P) characteristic peaks, indicating a certain amount of sulfides in the oil shale. Multiple distinct diffraction peaks, demonstrating that the minerals in the oil shale sample exhibit good crystallinity. No significant features for organic matter in the XRD spectrum, as organic matter is primarily amorphous.

The XRD spectrum of the semi-coke sample (Figure b) shows:

The quartz (Q) characteristic peaks remain prominent, and their intensity significantly increases, indicating that quartz becomes relatively enriched during the pyrolysis process. The calcite (C) characteristic peaks weaken considerably, indicating that under high temperature conditions, calcite may undergo decomposition to produce CO_2_, which is consistent with its known thermal decomposition characteristics. The pyrite (P) characteristic peaks also weaken, indicating that some sulfides may have decomposed or transformed. New potassium feldspar (K) characteristic peaks appear in the semi-coke sample, which may result from the recrystallization of minerals during high-temperature reactions. It is important to note that the changes in XRD peak intensity reflect relative crystallinity rather than absolute content.

High-temperature pyrolysis not only leads to the cracking of organic matter but also causes the decomposition, enrichment, and recrystallization of mineral components. These results highlight the profound impact of pyrolysis on the solid-phase composition of oil shale.

**Fig. 7:**
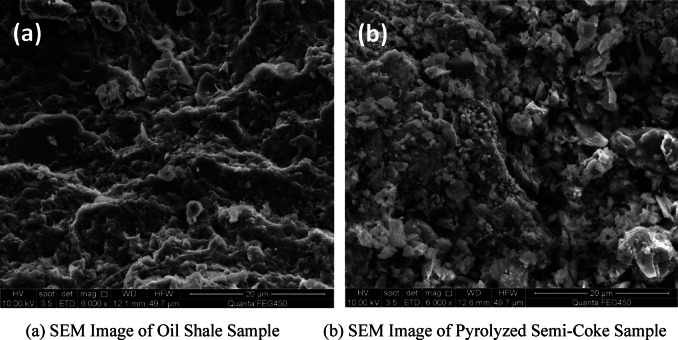
SEM images of oil shale and semi-coke samples.

From the comparison analysis of Fig. 7 between the SEM images of oil shale and semi-coke samples, it is evident that under high-temperature conditions, the porosity of the residue surface increases, and the microstructure exhibits a porous characteristic. Temperature has a significant impact on the properties of the semi-coke. High temperatures not only promote the decomposition of minerals but also enhance the porous structure of the residue, making it more suitable for resource utilization in the field of construction materials.

**Fig. 8:**
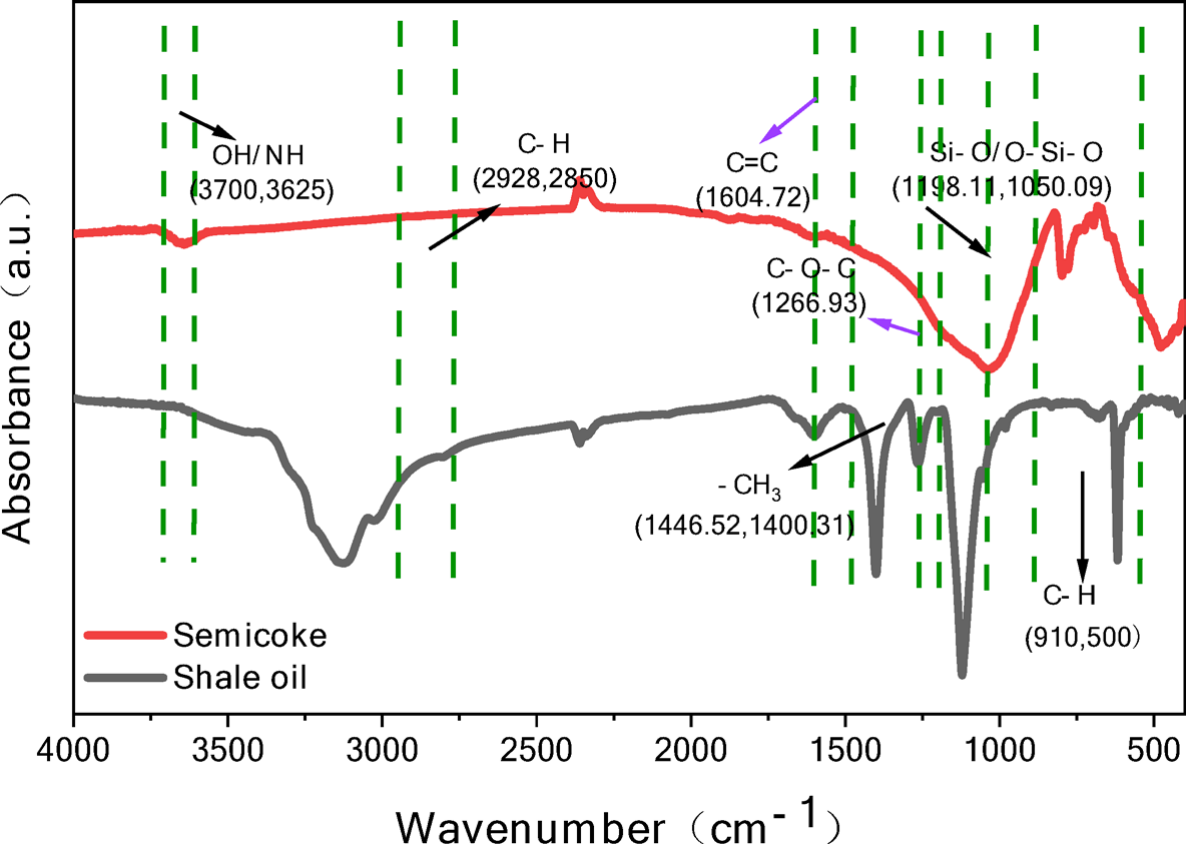
Infrared spectra of oil shale pyrolysis products.

Figure 8 shows the infrared spectra of pyrolysis products (shale oil and semi-coke) from oil shale.

C–H stretching vibration (2928, 2850 cm⁻^1^): Significant C–H absorption peaks appear in shale oil, indicating the presence of a large amount of methyl and methylene structures in alkanes and aromatic hydrocarbons. These peaks are weaker in semi-coke, suggesting that C–H bonds in the organic matter are partially broken during pyrolysis, resulting in the generation of gaseous and liquid hydrocarbons.

C=C double bond stretching vibration peaks (1604, 1600 cm^−1^): Shale oil exhibits noticeable C=C absorption peaks, indicating the presence of aromatic hydrocarbons or unsaturated compounds in shale oil. In semi-coke, the C=C peaks almost disappear, showing that most unsaturated bonds have been transformed or polymerized during high-temperature pyrolysis.

C=O stretching vibration peak (1266 cm^−1^): Shale oil contains C=O functional groups, which may represent characteristic peaks of organic acids, esters, or ketones. This peak weakens in semi-coke, indicating that oxygen-containing groups are gradually removed under high-temperature conditions.

Si–O–Si vibration peaks (1198, 1050 cm^−1^): Strong Si–O–Si absorption peaks are observed in semi-coke, primarily related to the mineral components (e.g., quartz) in oil shale. These peaks are weaker in shale oil, indicating that shale oil is mainly derived from organic matter, while the mineral residues remain in the semi-coke.

C–H bending vibration peaks (1446, 1408 cm^−1^ and 910, 500 cm^−1^): These absorption peaks are prominent in shale oil, reflecting the presence of methyl, methylene, and hydrocarbon skeletons. In semi-coke, these peaks weaken significantly, indicating that most hydrocarbons are converted into oil or gas during pyrolysis.

The infrared spectrum of shale oil exhibits strong C–H stretching and bending vibration peaks, indicating that its main components are alkanes and aromatic hydrocarbons. The presence of functional groups such as O–H, C=C, and C=O suggests that shale oil contains a certain amount of oxygenated and unsaturated compounds. In contrast, the infrared spectrum of semi-coke shows a significant reduction in the intensity of peaks corresponding to organic groups like C–H and C=O, indicating that the organic matter undergoes cracking during pyrolysis.

Infrared spectral analysis reveals that shale oil primarily consists of hydrocarbons, with a high content of alkanes and a small amount of oxygenated compounds. In semi-coke, organic groups are significantly reduced, and mineral components dominate. This indicates that the pyrolysis process decomposes the organic matter in oil shale into liquid oil and gas, leaving semi-coke enriched with minerals.

## Mechanism analysis

The pyrolysis process of oil shale is heavily influenced by temperature, which determines the reaction pathways and the formation of products. Based on experimental results and reaction kinetics analysis, the pyrolysis mechanism of oil shale is discussed from three aspects: decomposition stages, reaction pathways, and the regulatory role of temperature.

### Decomposition stages of pyrolysis

According to the characteristic temperatures and product distribution during the pyrolysis process, the pyrolysis of oil shale can be divided into the following three stages^13–16^:

1. Low-Temperature Dehydration Stage (< 200 °C):

In this stage, physical dehydration occurs as adsorbed and bound water in the sample evaporates. There is minimal mass loss, and no significant chemical reactions take place.

(2) Medium-Temperature Organic Matter Cracking Stage (400–650 °C):

The organic matter in oil shale (e.g., kerogen) begins to undergo thermal cracking, generating volatile components. This stage is the main reaction phase, where significant amounts of liquid products (shale oil) and combustible gases (e.g., CO_2_, CH_4_) are released. The reactions primarily involve the breaking of carbon chains and the removal of functional groups, such as decarboxylation reactions (producing CO_2_) and methyl cracking (producing CH_4_).

(3) High-Temperature Recombination and Residue Decomposition Stage (> 650 °C):

At high temperatures, the heavier components formed during cracking undergo further decomposition, generating additional gaseous products (e.g., H_2_, CO). Meanwhile, the mineral matter in the semi-coke (e.g., carbonates) thermally decomposes to form oxides, and the fixed carbon is converted into coke.

### Analysis of pyrolysis reaction pathways

Based on the analysis of liquid and gaseous products, the primary reaction pathways of oil shale pyrolysis are proposed as follows^17^:

1. Primary Cracking Reactions:
  $${\text{Organic}}\;{\text{ matter }}\left( {{\text{kerogen}}} \right) \, \to {\text{ Shale}}\;{\text{ oil }} + {\text{ Gas }} + {\text{ Semi - coke}}$$

During the medium-temperature stage, kerogen decomposes into tar and volatile components, while releasing gases such as CH_4_ and CO_2_. This reaction is primarily driven by the cleavage of chemical bonds with lower activation energy, such as weak C–C and C–H bonds.

(2) Secondary Cracking Reactions:
  $${\text{Shale }}\;{\text{oil }} \to {\text{ Gas }}\left( {{\text{CH}}_{{4}} ,{\text{ H}}_{{2}} ,{\text{ etc}}.} \right) \, + {\text{ Solid }}\;{\text{carbonaceous}}\;{\text{ residue}}$$

Under high-temperature conditions, the heavier components in shale oil undergo further cracking, increasing the proportion of lighter components (e.g., ketones, ethers), while simultaneously producing gases.

(3) Residue Mineral Decomposition:
  $${\text{Minerals }}\left( {{\text{e}}.{\text{g}}.,{\text{ carbonates}}} \right)\, \to \,{\text{Oxides }}\left( {{\text{e}}.{\text{g}}.,{\text{ CaO}},{\text{ MgO}}} \right)\, + \,{\text{CO}}_{{2}}$$

At high temperatures, the carbonate minerals in the semi-coke decompose, forming porous oxide structures.

### Regulatory role of temperature in reaction pathways

Temperature significantly affects the reaction rate, pathway selection, and product distribution in oil shale pyrolysis:

1. Regulation of Gas Product Pathways:

High-temperature conditions (> 600 °C) enhance secondary cracking reactions, promoting the production of light gases (H_2_, CH_4_). As the temperature increases, gas yield rises, and the proportion of CO and H_2_ increases, indicating that high temperatures favor the generation of gaseous products and improve the calorific value of the gas.

2. Regulation of Liquid Product Pathways:

Medium-temperature conditions (400–600 °C) represent the optimal range for tar generation. During this phase, primary cracking reactions dominate, resulting in the highest shale oil yield. At higher temperatures, shale oil undergoes secondary cracking into gases, leading to a reduction in liquid products.

3. Regulation of Semi-coke Pathways:

Increasing temperatures promote mineral decomposition and carbonization reactions, reducing the fixed carbon content in the residue and increasing its porosity.

### Kinetic analysis

The kinetic analysis of oil shale pyrolysis was conducted using the FWO (Flynn–Wall–Ozawa) and KAS (Kissinger–Akahira–Sunose) methods. These approaches comprehensively characterize the variation of activation energy during the pyrolysis process, providing a theoretical basis for optimizing the pyrolysis process and improving oil yield^18–20^.

**Fig. 9:**
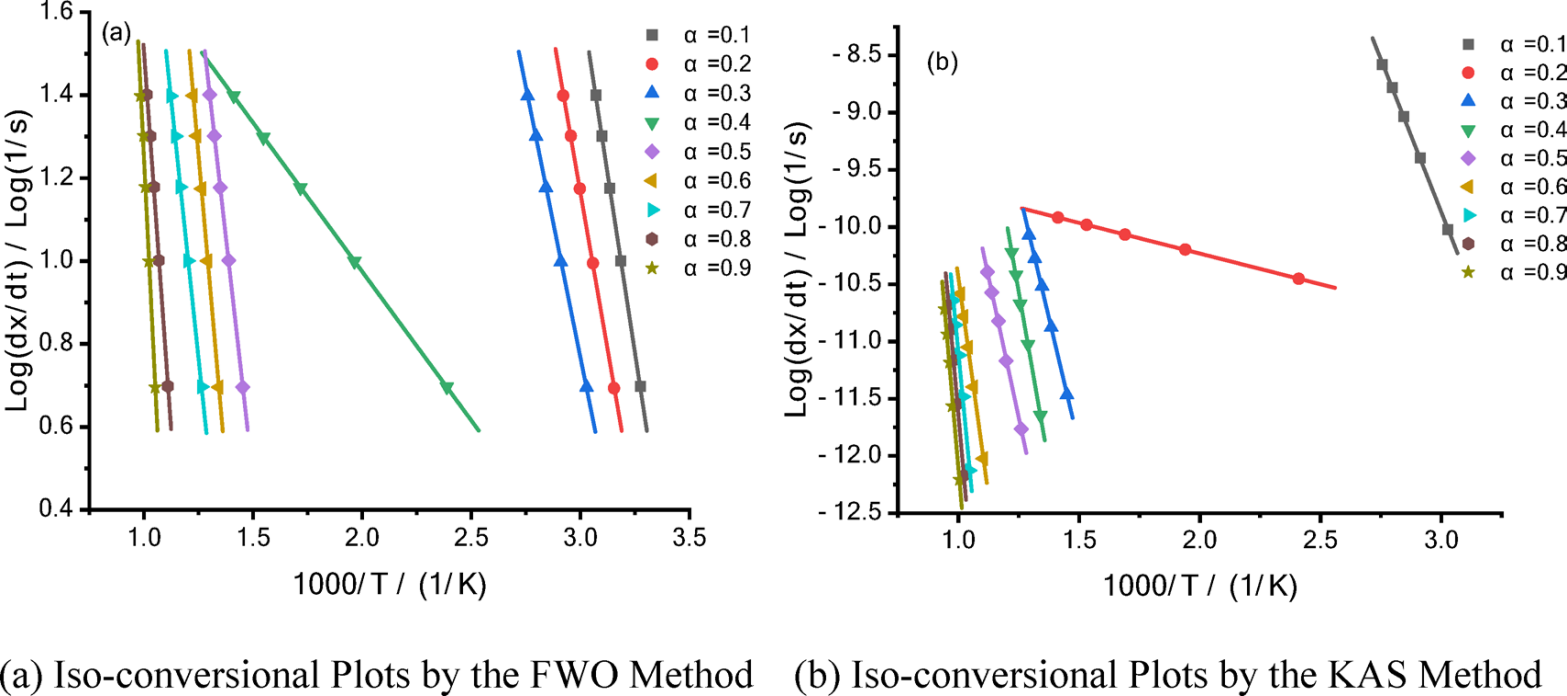
Kinetic Parameter Calculations for Oil Shale Pyrolysis Based on the FWO and KAS Methods.

In experiments conducted at different heating rates, the relationship between Log(dx/dt) and 1000/T at the same conversion rate is shown in Fig. 9.

From Fig. 9, the following can be observed:

Initial Stage (low conversion rate α = 0.1–0.3): The slope is steep, and the activation energy is high. This stage likely corresponds to the initial decomposition of volatile substances.

Intermediate Stage (moderate conversion rate α = 0.4–0.6): The slope gradually decreases, and the activation energy lowers, indicating the main reaction phase of organic matter decomposition.

Final Stage (high conversion rate α = 0.7–0.9): The slope becomes nearly flat, and the activation energy reaches its lowest point. This stage likely corresponds to deep cracking and the formation of semi-coke.

The FWO method analyzes the pyrolysis reaction through non-isothermal processes, providing insights into the variation of activation energy with conversion rates. It is particularly suitable for analyzing activation energy during the initial and intermediate stages of the reaction.

The KAS method calculates the reaction activation energy more directly and is applicable to the entire pyrolysis process. It is especially useful for estimating energy requirements during high conversion rates (deep cracking stage).

In kinetic analysis, the non-isothermal reaction rate equation is derived by combining the Arrhenius equation with the law of mass action. Accordingly, the kinetic analysis of oil shale samples was performed. Table 3 presents the kinetic data calculated using the FWO and KAS model-free methods.

**Table 3:** Kinetic data for oil shale pyrolysis calculated using the FWO and KAS model-free methods.

Conversion rate (α)	0.1	0.3	0.5	0.7	0.9
Activation energy Ea (kJ/mol) by FWO method	44.54	74.25	82.16	178.91	198.28
Activation energy Ea (kJ/mol) by KAS method	44.44	73.94	81.80	178.73	198.09

From Table 3, the following conclusions can be drawn:

Low Conversion Rate Stage (α = 0.1–0.3): The activation energy is relatively low, indicating that the initial stage of pyrolysis primarily involves the release of volatiles such as moisture and light organic compounds. This phase is characterized by reactions that are relatively facile and require minimal energy, consistent with the attributes of primary pyrolysis reactions.

Moderate Conversion Rate Stage (α = 0.5–0.7): There is a notable increase in the activation energy, signifying that the pyrolysis process has entered the main cracking phase of organic matter. During this stage, complex chemical bonds, such as C–C and C–H bonds within aromatic compounds, begin to break, necessitating higher energy input to sustain the reactions. This process predominantly entails the cracking of heavy organic compounds, leading to the formation of liquid products like shale oil and gaseous products such as CH_4_ and H_2_.

High Conversion Rate Stage (α = 0.9): The activation energy increases further, indicating that the latter stages of pyrolysis involve the decomposition of refractory materials, including aromatic compounds and coke precursors. These substances require significantly higher energies for their breakdown, resulting in a marked elevation in activation energy. This phase is characterized by the formation of semi-coke and a continued generation of gaseous products.

For the mechanism function, a G(α)−1/β linear fit was performed. Based on the analysis, the fit result should be a straight line passing through the origin, with the best linear correlation coefficient. When the intercept approaches 0, the corresponding G(α) is identified as the most probable mechanism function. Table 4 present the kinetic data results for four multi-step models.

**Table 4:** Multi-step model kinetic data for oil shale pyrolysis.

Model	Stage	Contribution	Reaction order (n)	Ea (kJ/mol)	logA (Log(1/s))	R^2^
Fn-Fn-Fn	Stage 1	0.464	2.361	5.728	− 2.347	0.993
	Stage 2	0.272	0.527	83.054	2.612	
	Stage 3	0.282	0.010	143.505	4.875	
An-D3-R3	An	0.487	0.584	13.171	− 2.428	0.988
	D3	0.223	–	19.792	− 0.672	
	R3	0.289	–	207.312	7.780	
D3-An-R3	D3	0.458	–	25.168	− 2.410	0.992
	An	0.233	0.133	8.463	− 1.823	
	R3	0.313	–	228.926	9.469	
R3-D3-An	R3	0.410	–	4.908	− 3.384	0.978
	D3	0.133	–	7.554	− 0.459	
	An	0.459	2.489	269.833	10.877	

This study employs multi-step kinetic models (such as Fn-Fn-Fn, An-D3-R3, etc.) to analyze the pyrolysis of oil shale. Oil shale pyrolysis encompasses multiple stages, including dehydration, organic matter cracking, and mineral decomposition, each characterized by distinct reaction mechanisms. A single model is inadequate for fully describing this complexity, whereas a multi-step model can more accurately reflect the characteristics of each stage. Although the Distributed Activation Energy Model (DAEM) is effective in describing activation energy distributions, it lacks the flexibility required for handling multi-stage reactions and is better suited for single reaction pathways. Given the multiplicity of reaction paths inherent in oil shale pyrolysis, multi-step kinetic models are more applicable and provide a more precise representation of the process dynamics.

From the kinetic analyses of the aforementioned four different models, the following conclusions can be drawn:

1. Fn-Fn-Fn Model.

The pyrolysis process is divided into three stages (Step 1, Step 2, Step 3), with the activation energy progressively increasing: 5.728 kJ/mol → 83.054 kJ/mol → 143.505 kJ/mol.

Step 1 contributes the most (46.4%), indicating that the initial reaction dominates. Low-temperature stage (Step 1), Activation energy is low, and the reaction proceeds easily, likely involving the initial release of volatile components in the organic matter. Medium-temperature stage (Step 2), Activation energy increases, indicating more complex reactions, such as the formation of intermediate products. High-temperature stage (Step 3), Activation energy reaches 143.505 kJ/mol, suggesting that deep cracking reactions require higher energy.

(2) An-D3-R3 Model.

This model combines nucleation and growth (An), diffusion (D3), and reaction control (R3) mechanisms. The contributions of each stage are as follows: 48.7% (An) → 22.3% (D3) → 28.9% (R3).

Nucleation and growth (An), The activation energy is the lowest (13.171 kJ/mol), suitable for the initial cracking of organic matter.

Diffusion control (D3), The activation energy is moderate (19.792 kJ/mol), indicating that the reaction is influenced by the diffusion of volatile components.

Reaction control (R3), The activation energy is higher (207.312 kJ/mol), indicating that deep cracking processes require overcoming significant energy barriers.

(3) D3-An-R3 Model.

The pyrolysis process involves three stages: diffusion control (D3), nucleation and growth (An), and reaction control (R3). The activation energy increases progressively: 25.168 kJ/mol (D3) → 228.926 kJ/mol (R3), reaching a maximum of 269.833 kJ/mol (An) in the high-temperature stage.

Diffusion control (D3), At the low-temperature stage, the reaction rate is primarily influenced by diffusion.

Nucleation and growth (An), At high temperatures, complex gas decomposition occurs, requiring high activation energy.

Reaction control (R3), Complex cracking reactions dominate, requiring the highest activation energy.

(4) R3-D3-An Model.

This model follows the sequence of reaction control (R3) → diffusion control (D3) → nucleation and growth (An). The activation energy progressively increases: 7.554 kJ/mol (D3) → 269.833 kJ/mol (R3) → 269.833 kJ/mol (An).

Key control factors in the kinetic process: Low-temperature stage: Dominated by diffusion control and nucleation. High-temperature stage: Dominated by complex chemical reactions.

Application of Multi-Step Kinetic Models: The multi-step kinetic model helps optimize the operational parameters of oil shale pyrolysis, control the pyrolysis reaction pathways, and improve shale oil yield and quality.

Insights into the Pyrolysis Process of Oil Shale: The pyrolysis of oil shale is a complex multi-stage, multi-path reaction system. Temperature regulation is a critical factor influencing cracking pathways and product distribution:

Medium-temperature stage: Dominated by primary cracking, making it ideal for shale oil production. High-temperature stage: Enhances secondary cracking and mineral decomposition, favoring the generation of gaseous products and highly porous residues.

This study’s mechanistic analysis provides theoretical support for optimizing the pyrolysis process, especially in temperature regulation. It offers guidance for achieving targeted product conversion and efficient resource utilization.

## Conclusion

This study systematically investigated the effects of temperature on product distribution and reaction mechanisms during oil shale pyrolysis using thermogravimetric analysis (TG) and fixed-bed experiments. While the findings provide valuable insights, certain limitations and practical considerations must be acknowledged to guide future research and industrial applications. The key conclusions are as follows:

1. The pyrolysis process can be divided into three stages: Low-temperature stage (< 200 °C): Dominated by moisture evaporation. Medium-temperature stage (400–650 °C): Primary decomposition of organic matter, yielding maximal shale oil. High-temperature stage (> 650 °C): Secondary cracking of heavy components and mineral decomposition, favoring gas production. However, the exact transition temperatures may vary with shale composition, necessitating further validation across diverse samples.
2. Gas yield increased significantly at high temperatures, with H_2_ and CH_4_ becoming dominant. Shale oil yield peaked at 400–500 °C but declined above 550 °C due to secondary cracking. Semi-coke porosity improved at higher temperatures, but its yield decreased. While high temperatures enhance gas production, the associated energy costs and trade-offs in liquid yield must be evaluated for economic viability.
3. Medium temperatures favored primary cracking (kerogen → shale oil), while high temperatures promoted secondary reactions (shale oil → gases). Mineral decomposition (e.g., carbonates → oxides) further contributed to gas release. Kinetic analysis revealed multi-stage mechanisms, but the models’ applicability to industrial-scale processes requires further testing.
4. For shale oil production, temperatures of 400–500 °C are optimal, balancing yield and energy input. For gas production, temperatures > 650 °C are recommended, though energy efficiency and downstream gas purification costs should be considered. Semi-coke utilization (e.g., adsorbents, construction materials) is viable but depends on mineral composition and pyrolysis conditions. Future studies should address scalability, real-world energy inputs, and the impact of shale heterogeneity on product quality.

This study elucidates the key mechanisms by which temperature affects the distribution of pyrolysis products in oil shale and provides scientific guidance for optimizing pyrolysis processes. The findings also offer important insights for the efficient development and resource utilization of unconventional energy sources.

## Data Availability

All the data generated or analyzed during this study are included in this published article (in Figures and Tables). The datasets are also available from the corresponding author on reasonable request.

## References

[1] Li, Y. H. et al. The present situation and progress of oil shale exploration and exploitation. *CT Theory Appl.* **23** (6), 1051–1063 (2014).

[2] Zhang, H. G., Zhao, Y. S., Yang, D. & Wang, L. Study on the effect of temperature on the Pyrolysis-Mechanics-Seepage characteristics of oil shale. *J. Taiyuan Univ. Technol.* **52** (6), 945–952 (2021).

[3] Wang, L., Yang, D. & Kang, Z. Q. Experimental study on permeability characteristics and anisotropy evolution of oil shale after high-temperature water vapor treatment. *Chin. J. Rock Mechan. Eng.* **40** (11), 2286–2295 (2021).

[4] Tissot, B. P. & Welte, D. H. *Petroleum Formation and Occurrence* (Springer Science & Business Media, 2013).

[5] Lin, L., Lai, D., Shi, Z., Han, Z. & Xu, G. Distinctive oil shale pyrolysis behavior in indirectly heated fixed bed with internals. *RSC Adv.* **7** (35), 21467–21474 (2017).

[6] Al-Ayed, O. S., Al-Harahsheh, A., Khaleel, A. M. & Al-Harahsheh, M. Oil shale pyrolysis in fixed-bed retort with different heating rates. *Oil Shale* **26**(2), 139–147 (2009).

[7] Kok, M. V. & Pamir, R. Pyrolysis kinetics of oil shales determined by DSC and TG/DTG.*Oil Shale* **20**(1), 57–68 (2003).

[8] Kok, M. V. Oil shale: Pyrolysis, combustion, and environment: A review. *Energy Sources*. **24** (2), 135–143 (2002).

[9] Abu El-Rub, Z., Kujawa, J. & Al-Gharabli, S. Pyrolysis kinetic parameters of Omari oil shale using thermogravimetric analysis. *Energies* **13** (16), 4060 (2020).

[10] Bai, F., Sun, Y., Liu, Y., Li, Q. & Guo, M. Thermal and kinetic characteristics of pyrolysis and combustion of three oil shales. *Energy. Conv. Manag.* **97**, 374–381 (2015).

[11] Skala, D., Kopsch, H., Sokić, M., Neumann, H. J. & Jovanović, J. A. Kinetics modelling oil shale pyrolysis. *Fuel* **69**(4), 490–496. (1990).

[12] Na, J. G., Im, C. H., Chung, S. H. & Lee, K. B. Effect of oil shale retorting temperature on shale oil yield and properties. *Fuel* **95**, 131–135 (2012).

[13] Amer, M. W., Alhesan, J. S. A., Marshall, M., Awwad, A. M. & Al-Ayed, O. S. Characterization of Jordanian oil shale and variation in oil properties with pyrolysis temperature. *J. Anal. Appl. Pyrol.* **140**, 219–226 (2019).

[14] Palayangoda, S. S. & Nguyen, Q. P. Thermal behavior of raw oil shale and its components. *Oil Shale*. **32** (2), 160 (2015).

[15] Al-Harahsheh, M. et al. Effect of demineralization and heating rate on the pyrolysis kinetics of Jordanian oil shales. *Fuel Process. Technol.* **92** (9), 1805–1811 (2011).

[16] Liu, X. et al. Initial pyrolysis mechanism of oil shale kerogen with reactive molecular dynamics simulation. *Energy Fuels* **29** (5), 2987–2997 (2015).

[17] Qing, W., Xinmin, W. & Shuo, P. Study on the structure, pyrolysis kinetics, gas release, reaction mechanism, and pathways of Fushun oil shale and kerogen in China. *Fuel Process. Technol.* **225**, 107058 (2022).

[18] Zhang, J., Ding, Y., Du, W., Lu, K. & Sun, L. Study on pyrolysis kinetics and reaction mechanism of Beizao oil shale. *Fuel* **296**, 120696 (2021).

[19] Chang, Z. et al. Influence of inherent mineral matrix on the product yield and characterization from Huadian oil shale pyrolysis. *J. Anal. Appl. Pyrol.* **130**, 269–276 (2018).

[20] Torrente, M. C. & Galan, M. A. Kinetics of the thermal decomposition of oil shale from Puertollano (Spain). *Fuel* **80** (3), 327–334 (2001).

